# Promotion or Suppression of Murine Intestinal Polyp Development by iNKT Cell Directed Immunotherapy

**DOI:** 10.3389/fimmu.2019.00352

**Published:** 2019-03-01

**Authors:** Ying Wang, Saikiran K. Sedimbi, Linda Löfbom, Gurdyal S. Besra, Steven A. Porcelli, Susanna L. Cardell

**Affiliations:** ^1^Department of Microbiology and Immunology, Institute of Biomedicine, University of Gothenburg, Gothenburg, Sweden; ^2^School of Biosciences, Institute of Microbiology and Infection, University of Birmingham, Birmingham, United Kingdom; ^3^Department of Microbiology and Immunology, and Department of Medicine, Albert Einstein College of Medicine, Bronx, NY, United States

**Keywords:** NKT cells, tumor immunotherapy, intestine, α-galactosylceramide, CD1d

## Abstract

The glycosphingolipid α-galactosylceramide (α-GalCer) is a well-described immune activator with strong anti-tumor properties in animal models. It is presented on CD1d and acts by stimulating the invariant, type I, natural killer T (iNKT) lymphocytes to rapidly secrete TH1 and TH2 associated cytokines. This in turn promotes activation of a diversity of immune cells including natural killer (NK) cells with anti-tumor functions. Prior to tumor development, iNKT cells can also perform tumor surveillance and naturally protect from emergence of cancer. In contrast, we have recently demonstrated that iNKT cells naturally promote polyps in the spontaneous murine adenomatous polyposis coli (Apc) *Apc*^*Min*/+^ model for colon cancer, associated with suppressed TH1 immunity and enhanced immunoregulation. Here we investigated whether iNKT cell directed immunotherapy could subvert the polyp promoting function of iNKT cells and reduce polyp growth in this model. We treated *Apc*^*Min*/+^ mice with α-GalCer, or synthetic derivatives of this ligand (C-glycoside and C20:2) that have enhanced immunoregulatory properties. Treatment with iNKT cell ligands led to increased iNKT cell division, but reduced iNKT cell frequencies, lower NK1.1 expression and elevation of PD-1. *Apc*^*Min*/+^ mice that had been treated either long-term (5–15 weeks of age), or short-term (12–15 weeks of age) with α-GalCer demonstrated a significant decrease in polyp burden. Surprisingly, long-term treatment with the TH1 biasing ligand C-glycoside did not have significant effects on polyps, while long-term treatment with the TH2 biasing ligand C20:2 enhanced polyp growth. In stark contrast, short-term treatment with C20:2 led to reduction in polyp numbers and size. Reduced polyp burden after long-term treatment was associated with increased expression of genes indicating a pro-inflammatory polyp microenvironment. Polyp-reducing short-term treatment led to CD8 T cell activation specifically in polyps, and decreased tumor infiltrating and splenic macrophages, and a switch toward a pro-inflammatory phenotype. Thus, iNKT cell directed therapy could subvert the natural polyp enhancing function of iNKT cells, overcome immunosuppression, and reduce polyps. However, different iNKT cell activating ligands had opposite effects, and the timing of treatment had a major influence on outcomes.

## Introduction

CD1d-restricted natural killer T (NKT) cells are activated by endogenous or exogenous lipid ligands presented on the essentially non-polymorphic MHC class I-like molecule CD1d. The high conservation between human and mouse CD1d, and CD1d-restricted NKT cells, and their strong cytokine mediated immunomodulatory effects, has prompted studies investigating NKT cell directed therapy in various immune settings ranging from autoimmunity to tumor immunity. The highly potent ligand α-galactosylceramide (α-GalCer), derived from a marine sponge, was originally identified in a screen for compounds that prevented lung metastasis in the B16 melanoma mouse model (1). Studies subsequently demonstrated that α-GalCer C26:0, also designated KRN7000, potently activates a subset of NKT cells called invariant NKT (iNKT) or type I NKT cells that expresses an invariant TCR Vα14 chain (2) (Vα24 in humans). The invariant TCRα chain pairs in most cases with Vβ8, 7 or 2 (Vβ11 in humans) TCR β-chains to generate a semi-invariant receptor that mediates selection and subsequent activation in a CD1d dependent manner (3–5). In contrast, another subset termed type II NKT cells expresses a more diverse TCR repertoire and responds to other ligands presented on CD1d (6–8).

In several mouse models, treatment with α-GalCer suppresses tumor development (9–12). In humans, iNKT cells are reduced in different cancers, and higher proportions of iNKT cells among tumor infiltrating immune cells has been associated with a favorable outcome (13–15), suggesting that increasing iNKT cell anti-tumor activity could be a useful approach. Thus, iNKT cell directed tumor immunotherapy is being further developed and translated into the clinic, and clinical trials show promising results (13–15). Stimulation of iNKT cells with α-GalCer can enhance their anti-tumor activities, and this has been linked to the rapid production of both pro- and anti-inflammatory cytokines. The development of structurally modified α-GalCer analogs have demonstrated that while α-GalCer stimulation of iNKT cells results in a mixed cytokine production including TH1-, TH2-, and TH17 associated cytokines, certain analogs of α-GalCer alter the cytokine profile produced by iNKT cells and provide enhanced therapeutic effects (10, 12). The synthetic C-glycoside analog α-C-galactosylceramide (C-glycoside) skews the cytokine response by iNKT cells toward TH1. C-glycoside has superior anti-metastatic effects compared to α-GalCer in tumor models, thought to result from prolonged induction of TH1 cytokine production and enhanced downstream activation of NK cells (16, 17). In contrast, α-GalCer with a shortened and di-unsaturated fatty acyl chain (C20:2) induced a TH2 biased cytokine profile with high amounts of IL-4 and reduced IFN-γ production (18), and afforded a stronger protection than α-GalCer from type I diabetes in non-obese diabetic mice (19). Thus, these ligands provide more sophisticated tools for iNKT cell directed therapy, and may be particularly relevant for situations when a specific cytokine profile is desired.

Mutations in the human adenomatous polyposis coli (*Apc*) gene cause hereditary colon cancer, and are present in a majority of sporadic colorectal cancers (20). The *Apc*^*Min*/+^ mice carry a truncated *Apc* gene, a dominant mutation that leads to the spontaneous formation of intestinal adenomas in heterozygote mice, recapitulating early events in human colorectal carcinogenesis (21–23). Inflammation is an established driving force in many cancers including colon cancer (24), and studies have also demonstrated the essential role of inflammatory signals driving the formation of polyps in *Apc*^*Min*/+^ mice (25). In contrast, a TH1-type immune response is regarded as protective (26, 27). We have recently demonstrated that iNKT cells promoted polyp development in *Apc*^*Min*/+^ mice (28). Absence of iNKT cells strongly reduced the number of polyps and was associated with an increase in the expression of TH1 associated genes in polyps and lamina propria, a decrease in the frequency of T regulatory cells (T_reg_) specifically in polyp tissue, and a shift from an M2- to an M1-like macrophage phenotype. This suggests that iNKT cells naturally suppressed TH1 tumor immunity in this model by promoting T regulatory cells and M2 macrophages (28, 29).

In the present study, we have investigated whether iNKT cell directed immunotherapy could subvert the natural tumor promoting function of iNKT cells and reduce polyp development in the spontaneous orthotopic *Apc*^*Min*/+^ mouse model for colon cancer. To this end, we have taken advantage of α-GalCer and its structural analogs, the TH1 skewing C-glycoside and TH2 skewing C20:2, and performed preclinical studies in the *Apc*^*Min*/+^ mouse model. We demonstrate that iNKT cell targeted tumor immunotherapy can reduce polyp development, however, the timing of treatment, and choice of ligand were essential for treatment effects.

## Materials and Methods

### Mouse Strains and Breeding

The *Apc*^*Min*/+^ mutation is bred on the C57BL/6 genetic background. The *Apc*^*Min*/+^ breeding was maintained by crossing male *Apc*^*Min*/+^ mice with female *Apc*^+/+^ mice, and *Apc*^*Min*/+^ females were used in most experiments. We did not observe any difference in polyp numbers in male and female *Apc*^*Min*/+^ mice (data not shown). All mice were bred and maintained at the department of Experimental Biomedicine, University of Gothenburg. This study was carried out in accordance with the national animal ethics regulations. The protocol was approved by the animal ethics committee in Gothenburg (Göteborgs djurförsöksetiska nämnd).

### Long-Term *in vivo* Treatment With Glycolipid

**Figure 1:**
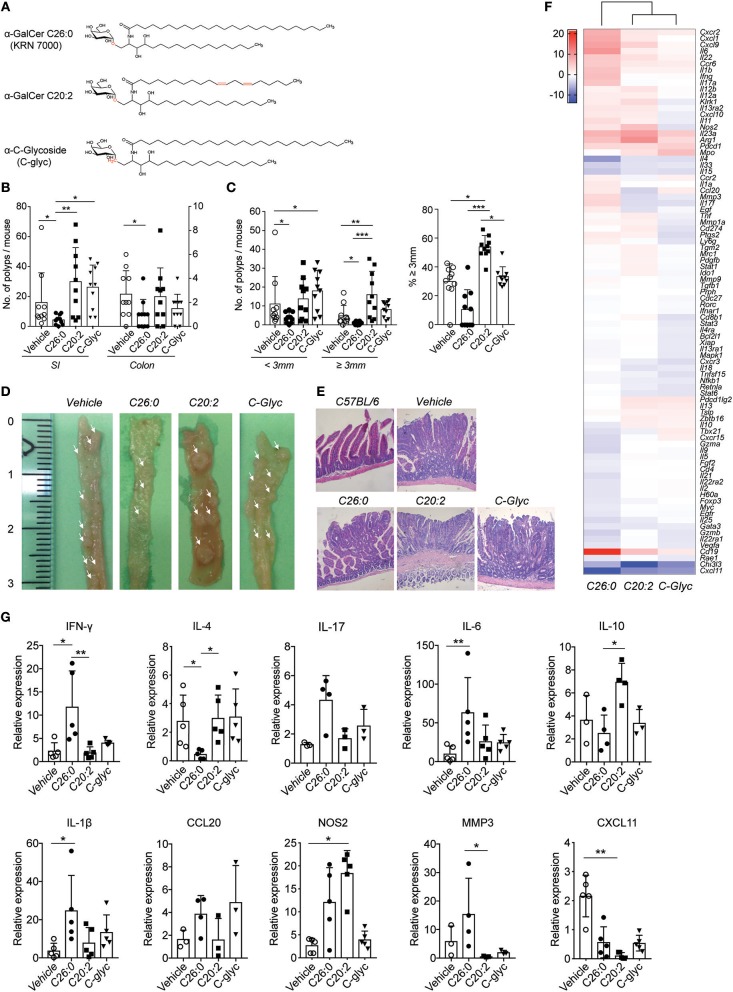
Long-term treatment with α-GalCer C26:0 and modified α-GalCer analogs differentially modulated polyp development in *Apc*^*Min*/+^ mice. **(A)** Structure of α-GalCer C26:0, C20:2, and C-glycoside. Mice were treated from 5 weeks of age as described in Materials and Methods, and sacrificed at 15 weeks of age. Polyps from small intestine (SI) and colon were counted **(B)**. **(C)** The number of small (<3 mm) and large (≥ 3 mm) polyps in SI was counted (left graph) and the proportion of large polyps was calculated (right graph). The data are presented as mean ± SD of 10 mice. Kruskal-Wallis test, corrected for multiple comparisons using Dunn's test, was used for statistical analyses. ^*^p < 0.05, ^**^p < 0.01, ^***^p < 0.001. **(D)** The small intestines were photographed, left scale indicates centimeters. Macroscopic polyps in small intestines are indicated by arrows. **(E)** The small intestines were isolated and fixed in paraformaldehyde, and the tissues were sectioned and stained with hematoxylin/eosin. Tissues from representative mice are shown. **(F)** Heat map of the expression of selected genes in the polyp tissue. Total mRNA was isolated from polyp tissue of treated mice. The expression of mRNA was examined by RT^2^ profiler PCR array with a selection of genes of relevance for immunity and tumor progression. Each sample was a pool of mRNA from 5 mice and was run in duplicate. CT values are provided in Supplementary Table 1. The heatmap shows gene expression in polyps from ligand treated mice relative to polyps from vehicle treated mice, with vehicle expression values set to “0.” The scale bar indicates fold change expression to vehicle group. **(G)** The expression of selected genes was examined by real-time PCR and normalized against β-actin. Symbols represent individual mice and data are presented as mean ± SD of 3–5 mice. Kruskal-Wallis test, corrected for multiple comparisons using Dunn's test, was used for statistical analyses. ^*^p < 0.05, ^**^p < 0.01.

Lyophilized glycolipids [α-GalCer C26:0, α-GalCer C20:2 (18), α-C-glycoside (16)] (Figure 1A) were dissolved as described before (28). Mice were injected i. p. with 4 μg of glycolipid in 200 μl of PBS with a final concentration 0.1% DMSO, 0.05% Tween-20. Vehicle control was prepared and injected in an identical manner. Five week old female *Apc*^*Min*/+^ mice (10 in each group) were treated with α-GalCer C26:0, α-GalCer C20:2, α-C-glycoside or vehicle control on day 1, 2, 7, 14, 21, 28, and 60, and the mice were sacrificed at 15 weeks of age.

### Short-Term *in vivo* Treatment With Glycolipid

Lyophilized glycolipids (α-GalCer C26:0, α-GalCer C20:2) were dissolved in vehicle (PBS including 5.6% sucrose, 0.75% L-histidine, and 0.5% Tween-20), sonicated for 5 min and immediately heated at 80°C for 2 min in glass vials and kept in an 80°C bath until shortly before injection. Mice were injected i. p. with 4 μg of glycolipid in 200 μl of vehicle. Vehicle control was prepared and injected in an identical manner. 12 week old female *Apc*^*Min*/+^ mice (13 in each group) were treated with α-GalCer C26:0, α-GalCer C20:2, or vehicle control on day 1, 8, 15, and the mice were sacrificed at 15 weeks of age.

### Polyp Counting and Scoring

Mice were sacrificed at 15 weeks of age. The intestines were flushed with PBS from both sides using blunt end gavage needles to remove fecal material, and small intestines (SI) were cut into three equal length segments: duodenum, jejunum, and ileum. Each segment was then cut open longitudinally. Typical polyps were photographed when the intestines were open. Polyps were counted and scored by size (<3 mm or ≥3 mm).

### Organ Histology

Intestinal tissue was fixed with formalin at room temperature for 24 h. After processing, the formalin-fixed, paraffin-embedded (FFPE) tissues were vertically sectioned lengthwise. Tissue sections were stained with hematoxylin and eosin (H&E, performed by Histo-Center AB, Västra Frölunda, Sweden).

### RNA Isolation and RT^2^ Profiler PCR Array

Intestinal polyps from 15 week old *Apc*^*Min*/+^ mice were collected and saved in RNA later (Qiagen). Tissues were lysed and homogenized (Tissue lyser II, Qiagen) before total RNA was isolated using the High pure RNA tissue kit (Roche), including DNase I digestion. RNA concentration was determined spectrophotometrically with Nanodrop (ND-100, software version 2.5.1). The Quantitect reverse transcription kit (Qiagen) was used for cDNA synthesis, using 500 ng RNA as template in total reaction volume of 20 μl. The 384 (4 × 96) wells custom-made RT^2^ profiler PCR array (SA Bioscience, Qiagen) layout contained 4 replicate primer assays for each of 86 target genes and 7 housekeeping genes. In addition, 4 wells contained mouse genomic DNA controls, and 12 wells contained positive PCR controls. Prepared cDNA from pools of 5 mice were added in duplicates to RT^2^ SYBR Green Mastermix and the mix was aliquoted into the PCR array plates. PCR reactions were performed by the Genomics Core Facility, The Sahlgrenska Academy, and relative expression levels were determined using data from the real-time cycler and the ΔΔCT method. CT values of duplicates are provided in Supplementary Table 1. The heat map was made using Graphpad Prism 7, and shows fold change expression levels in polyps from ligand treated mice relative to vehicle treated mice, where vehicle levels are set to “0.”

### Quantitative PCR

RNA isolation and cDNA synthesis were performed as above. Each real time PCR mixture contained 40 ng cDNA, QuantiTect SYBR Green (Qiagen) and oligonucleotide primers to detect selected genes using β-actin as housekeeping gene. Primers were designed in Primer Express software version 2.1 (Applied Biosystems) and ordered from Sigma-Aldrich. Assays were run at standard thermal cycling conditions descried for 7,500 real-time PCR system (Applied Biosystems). The comparative CT method was used for gene expression analysis, in which β-actin served as endogenous control. The following primers were used for RT-PCR: β-actin forward (Fw) 5'- CTT CTT TGC AGC TCC TTC GTT-3′; β-actin reverse (Rev) 5′-AGG AGT CCT TCT GAC CCA TGC-3′; IFNγ Fw 5′-ACA ATG AAC GCT ACA CAC TGC-3′, IFNγ Rev 5′-CTT CCA CAT CTA TGC CAC TTG AG-3′; IL-4 Fw 5′-GGT CTC AAC CCC CAG CTA GT-3′, IL-4 Rev 5′-GCC GAT GAT CTC TCT CAA GTG AT-3′; IL-17 Fw 5′-CCG CAA TGA AGA CCC TGA TAG A-3′, IL-17 Rev 5′- TCA TGT GGT GGT CCA GCT TTC-3′; IL-6 Fw 5′- TAG TCC TTC CTA CCC CAA TTT CC-3′, IL-6 Rev 5′- TTG GTC CTT AGC CAC TCC TTC-3′; IL-10 Fw 5′-GCT CTT ACT GAC TGG CAT GAG-3′, IL-10 Rev 5′- CGC AGC TCT AGG AGC ATG TG-3′; IL1β Fw 5′-GGA TGA TGA TGA TAA CCT GC-3′, IL1β Rev 5′- CAT GGA GAA TAT CAC TTG TTG G-3′; CCL20 Fw 5′- GCT ATC ATC TTT CAC ACG AAG-3′, CCL20 Rev 5′- CAT CTT CTT GAC TCT TAG GC-3′; NOS2 Fw 5′- GTT CTC AGC CCA ACA ATA CAA GA-3′, NOS2 Rev 5′- GTG GAC GGG TCG ATG TCA C-3′; MMP3 Fw 5′- ACA TGG AGA CTT TGT CCC TTT TG-3′, MMP3 Rev 5′- TTG GCT GAG TGG TAG AGT CCC-3′.

### Lymphocyte Preparation

Spleen, mesenteric lymph nodes (MLN), SI and liver were collected from *Apc*^*Min*/+^ and *Apc*^+/+^ mice. Single cell suspensions from spleen and MLN were prepared by forcing the organs though nylon mesh using a syringe plunger. Liver lymphocytes were prepared by using Percoll as described before (28). Lamina propria (LP) lymphocytes and tumor infiltrating lymphocytes were isolated from the SI after removal of Peyer's patches. Polyp and unaffected LP tissue were separated from SI, and dissociated with Lamina Propria Dissociation Kit, Mouse (Miltenyi Biotec) by using gentelMACS dissociator (Miltenyi Biotec).

### Flow Cytometry

Tissues were processed, single cell suspensions were prepared and cells were stained. Fc block (Clone: 2.4G2) and vital dye (Live/Dead Fixable Aqua Dead Cell Stain, Invitrogen) were included in all the staining panels. Cells were stained with fluorescence-labeled antibodies obtained from BD Biosciences, eBioscience, and Biolegend. The following clones were used: CD3 (17A2), CD11b (M1/70), CD11c (N418), CD19 (1D3), CD4 (RMA4-5), CD8 (53-6.7), CD44 (IM7), CD45R/B220 (RA3-6B2), CD69 (H1.2F3), NK1.1 (PK136), CD199/CCR9 (CW-1.2), CD206 (C068C2), CD279/PD-1 (J43), FoxP3 (FJK-16s), F4/80 (BM8), iNOS (CXNFT), Ly6C (HK1.4), Ly6G (RB6-8C5), TCRβ (H57-597). PE and BV-421 labeled CD1d-tetramers loaded with α-GalCer (PBS57) were kindly provided by the NIH Tetramer Facility. Samples were acquired on an LSR-II flow cytometer (BD Bioscience) and analyzed using Flowjo software (Tree Star Inc.).

### Proliferation Assay

Lyophilized glycolipids were dissolved as described before (28) in a stock solution containing 1% DMSO, 0.5% Tween-20. Spleen cells (0.25 × 10^6^/well) were seeded in a 96 well-plate and incubated with or without 100 ng/ml lipid ligand at 37°C for 64 h. [^3^H]-thymidine was added to the wells and incubated for another 10–12 h followed by cell harvest. Incorporated radioactivity was counted in a MicroBeta counter (Perkin Elmer).

### Statistical Analyses

Calculation of statistical significance was performed using Kruskal-Wallis test, corrected for multiple comparisons with Dunn's post-tests. *P* < 0.05 were considered significant. Statistical analyses were performed on Prism GraphPad 7. Results are presented as mean ± SD in the figures.

## Results

### Effects of Long-Term Treatment With iNKT Cell Activating Ligands on Polyp Development

We first performed a long-term treatment schedule in *Apc*^*Min*/+^ mice starting at 5 weeks of age, around the time when polyps are initiated (30). Female *Apc*^*Min*/+^ mice were treated with synthetic α-GalCer (C26:0, KRN7000), the C20:2 analog biasing the cytokine response toward TH2, or the TH1 skewing analog C-glycoside (Figure 1A), according to the schedule described in Materials and Methods. Mice were sacrificed at 15 weeks of age. Treatment with the C26:0 form of α-GalCer resulted in a significant decrease in small intestine (SI) and colon polyp numbers (Figures 1B–E). Further, in the SI the numbers of both small and large (≥3 mm) polyps were significantly reduced compared to vehicle treated mice (Figure 1C). In contrast, mice treated with the TH2 skewing ligand C20:2 had significantly higher numbers of large SI polyps both compared to C26:0 and vehicle treated mice, while there was no effect in colon (Figures 1B,C). Polyps from C20:2 treated mice had a different appearance compared to other mice, with reddish circumference, and somewhat collapsed middle (Figures 1D,E). However, preliminary evaluation of hematoxylin and eosin stained sections suggests that all groups displayed intra-villous and sessile adenomas without invasion. Surprisingly, C-glycoside treatment also resulted in increased polyp numbers compared to C26:0 treated mice. The number of large polyps, however, was not significantly elevated, and the polyps had a macroscopic appearance similar to those in C26:0 and vehicle treated mice (Figures 1B–E). Histological examination of sections of polyps from the differently treated mice demonstrated grossly similar structure of the polyps without invasive behavior (Figure 1E). Thus, applying a long-term treatment procedure, we demonstrated that C26:0 treatment reduced both polyp number and size in SI, and polyp numbers in colon in the *Apc*^*Min*/+^ tumor model. In contrast, treatment with C20:2 enhanced polyp growth, whereas C-glycoside treatment did not result in significant effects on polyps compared to vehicle treatment.

### Suppression of Polyps Was Associated With a Distinct Gene Expression Signature

To investigate the tumor immune microenvironment in treated mice, we performed gene expression analysis of polyp tissue from the four groups of mice (Figure 1F, and Supplementary Table 1). We investigated a set of genes previously shown to include genes that were differently expressed in *Apc*^*Min*/+^ polyps in the presence or absence of iNKT cells, which was associated with high or low polyp burden, respectively (28). Polyps from mice treated with C26:0 compared to vehicle demonstrated an up-regulated expression of genes encoding IL-6, IFN-γ, IL-1β, IL17A, IL-17F, and MMP3. These genes were also more highly expressed in polyps from C26:0 treated mice compared both to polyps from C20:2 and from C-glycoside treated mice. In contrast, *Il4 and Il10* transcripts were found at higher levels in polyps from C20:2 and C-glycoside treated mice compared to polyps from C26:0 treated mice. While all ligand treatments compared to vehicle resulted in lower *Cxcl11* expression in polyps, C26:0 treatment induced higher expression levels of *Cxcl9, Cxcl1, Cxcr2*, and *Ccr6* compared to vehicle, suggesting increased immune cell recruitment to polyps after C26:0 treatment. This was not seen after C20:2 and C-glycoside treatment except for a lower induction of *Cxcl9* by C20:2. Gene expression in polyps from C20:2 compared to C-glycoside treated mice showed few differences, however, after C20:2 treatment a somewhat higher expression of *Nos2, Klrk1* (encoding NKG2D) and *Cd274* (encoding PD-L1), and lower expression of *Ccl20* was noted. All the above genes were altered 4-fold or more in the PCR expression array screen. qRT-PCR validation of a set of modulated genes largely confirms the PCR array data (Figure 1G). Taken together, this suggests that lower polyp burden after long-term treatment with C26:0 was associated with a pro-inflammatory TH1/TH17 associated tumor immune response.

### Long-Term Treatment With α-GalCer and Analog Ligands Resulted in Systemic Loss of iNKT Cells

**Figure 2:**
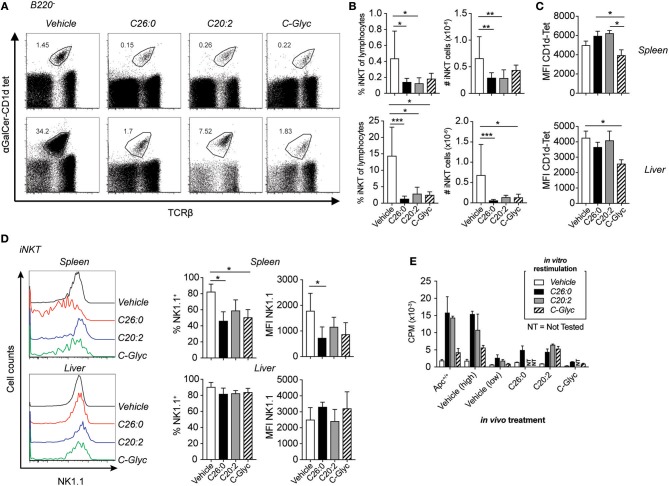
Long-term treatment of *Apc*^*Min*/+^ mice with α-GalCer and analogs resulted in systemic reduction of iNKT cells. C26:0 and the modified ligands C20:2, C-glycoside (C-Glyc), or vehicle were injected i. p. into *Apc*^*Min*/+^ mice as in Figure 1 starting at 5 weeks of age. At 15 weeks of age, mice were sacrificed and spleen or liver mononuclear cells were prepared and stained for flow cytometry. **(A)** B220^−^TCRβ^+^α-GalCer-CD1d tetramer^+^ cells were identified as iNKT cells. **(B)** Frequencies among total lymphocytes and absolute numbers of splenic (upper graphs) and liver (lower graphs) iNKT cells are shown for each group. **(C)** Median fluorescence intensity (MFI) of α-GalCer-CD1d tetramer in spleen (upper) and liver (lower) of each treatment group. **(D)** NK1.1 expression of iNKT cells was detected by flow cytometry. Representative histograms (left) and summary data of frequencies and MFI of NK1.1 expression on iNKT cells (right) are shown. Plots show profiles from representative mice **(A,D)** or is presented as mean ± SD of 10 mice **(B–D)**. Kruskal-Wallis test corrected for multiple comparisons using Dunn's test was used for statistical analyses. ^*^p < 0.05, ^**^p < 0.01, ^***^p < 0.001. **(E)** Spleen cells from treated mice were cultured with or without lipid ligands (100 ng/ml) and [^3^H]-thymidine incorporation was measured. Proliferation bar graph shows the response to different lipid ligands by individual mice, and are representative of 10 mice. Data are presented as mean ± SD of triplicate cultures. Vehicle treated mice demonstrated one of two different patterns, either high or low response to *in vitro* stimulation; an example of each is shown.

To determine the effects on iNKT cells of long-term treatment with the different ligands, we analyzed iNKT cells in treated mice by flow cytometry. iNKT cells were identified as TCRβ^+^ and α-GalCer-CD1d tetramer^+^ cells (Figure 2A). Long-term treatment led to a systemic reduction of frequencies and numbers of iNKT cells as detected in the spleen and liver in all groups compared to vehicle treated mice, as shown before, probably as a result of activation induced cell death (Figures 2A,B) (31–34). A lower level of α-GalCer-CD1d tetramer staining after C-glycoside treatment was found in the spleen (compared to C26:0 and C20:2) and in the liver (compared to vehicle treatment) (Figure 2C). There was a lower NK1.1 expression on the remaining splenic iNKT cells from ligand treated mice compared to vehicle treated mice (Figure 2D). This could be a consequence of activation-induced down modulation of NK1.1, or alternatively, selective survival of NK1.1^low^ iNKT cells. Splenocytes from 15 week old treated *Apc*^*Min*/+^ mice were stimulated *in vitro* with C26:0 or the respective ligands used for treatment (Figure 2E). C20:2 had similar stimulatory capacity to C26:0 *in vitro*, while C-glycoside stimulation resulted in lower proliferation when stimulating polyp free *Apc*^+/+^ littermate mice. Vehicle treated *Apc*^*Min*/+^ mice demonstrated two different patterns–some mice had a proliferative response similar to C57BL/6 (not shown) or *Apc*^+/+^ mice, while others showed a reduced response, possibly an effect of the polyp burden. The majority of ligand treated mice had a severely reduced *in vitro* proliferative response, consistent with the 2-3-fold reduced frequencies of splenic iNKT cells and an anergic state.

### Short-Term Treatment With C26:0 or C20:2 Suppressed Polyp Growth

At 12 weeks of age small polyps could be seen in the microscope on hematoxylin and eosin stained intestinal sections (28). At 15 weeks of age, polyps presented in all *Apc*^*Min*/+^ mice. This indicated that the period of polyp growth was most pronounced between 12 and 15 weeks. To investigate whether iNKT cell directed agonist treatment could modulate this late phase of polyp growth, we performed a short-term treatment schedule of *Apc*^*Min*/+^ mice starting at 12 weeks as described in Materials and Methods, and sacrificed the mice for analysis at 15 weeks of age as before. We excluded C-glycoside from the short-term treatment protocol as it had not shown positive effects using the long-term protocol. Thus, this time we compared mice treated with α-GalCer C26:0 and mice treated with C20:2, the two treatments that had resulted in significant but opposite effects on polyp burden using the long-term treatment protocol.

**Figure 3:**
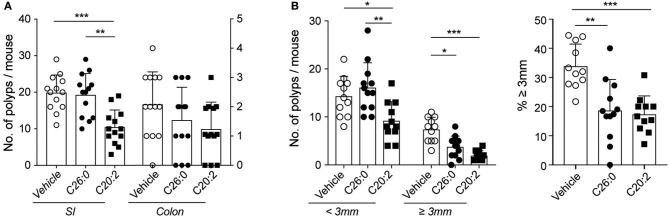
Short-term treatment with C26:0 and C20:2 suppressed polyp development in *Apc*^*Min*/+^ mice. Mice were treated with glycolipids from 12 weeks of age, as described in Materials and Methods. Mice were sacrificed at 15 weeks of age; polyps from SI and colon were counted **(A)** and the polyp size in SI was measured [**(B)**, left graph], and the proportion of large polyps calculated [**(B)**, right graph]. Data are presented as mean ± SD of 13 mice. Kruskal-Wallis test corrected for multiple comparisons using Dunn's test was used for statistical analyses. ^*^p < 0.05, ^**^p < 0.01, ^***^p < 0.001.

After short-term treatment of *Apc*^*Min*/+^ mice with C26:0 the numbers and proportion of large polyps in the SI were significantly reduced, but there were no effects on total colon and SI polyp numbers compared to vehicle treated mice (Figure 3). Surprisingly, short-term treatment with C20:2 had the reverse effect compared to long-term treatment with the same ligand. Total polyp numbers in SI as well as the number of large polyps, were significantly suppressed compared to vehicle treated mice (Figure 3). There was also a tendency of a reduction in colon polyps in C20:2 compared to vehicle treated mice, although the difference was not significant (Figure 3A). The somewhat higher polyp counts in the vehicle group after short-term treatment compared to long-term vehicle treatment is likely due to the use of different vehicles (see Materials and Methods). Thus, we demonstrate that short-term treatment with C20:2 resulted in a highly significant anti-tumor effect in the *Apc*^*Min*/+^ tumor model, while short-term treatment with C26:0 did not have as potent anti-tumor effect as in long-term treatment.

### Short-Term Treatment With α-GalCer (C26:0) and C20:2 Resulted in iNKT Cell Activation, Cell Loss and Anergic Phenotype

**Figure 4:**
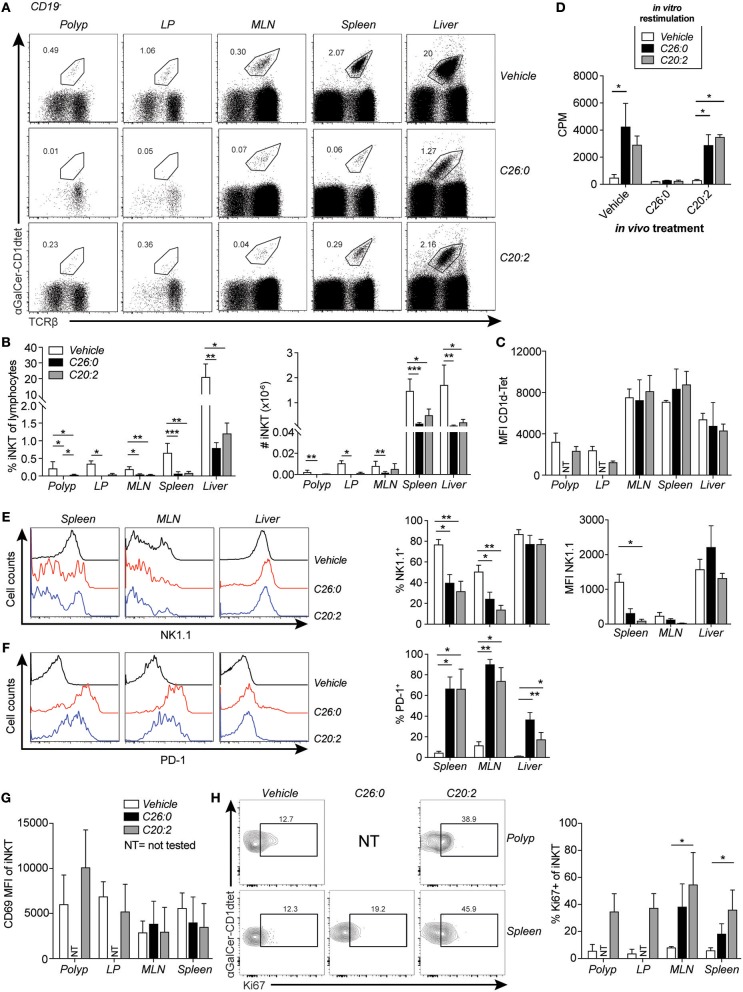
Short-term treatment of *Apc*^*Min*/+^ mice with C26:0 and C20:2 resulted in a reduction of iNKT cells and anergic phenotype, but different response to *in vitro* stimulation. Polyp, LP, MLN, spleen or liver mononuclear cells from mice treated as in Figure 3A were prepared and stained for flow cytometry. **(A)** Representative stainings of TCRβ^+^α-GalCer-CD1d tetramer^+^ iNKT cell populations after treatment. **(B)** Frequencies of iNKT cells among total lymphocytes and absolute number of iNKT cells are shown for each group. **(C)** MFI of α-GalCer-CD1d tetramer staining in the indicated organs of each group. Data are presented as mean ± SD of 3 (polyp, LP) to 10 mice (MLN, spleen and liver). **(D)** Spleen cells from treated mice were cultured for 3 days with or without lipid ligands (100 ng/ml) and [^3^H]-thymidine incorporation was measured during the last 10–12 h of culture. Data are presented as mean ± SD of triplicate cultures, and are representative of at least 5 mice per group. **(E)** Representative histogram and summary data of frequencies and MFI of NK1.1 expression on iNKT cells. **(F)** Representative histograms and summary data of frequencies of PD-1 expression on iNKT cells in the indicated organs after treatments. Data are presented as mean ± SD of 10 mice. **(G)** CD69 expression on iNKT cells in *Apc*^*Min*/+^ mice after treatments. **(H)** Representative staining of Ki67 on iNKT cells and frequencies of Ki67^+^ iNKT cells in indicated organs after treatments. Data are presented as mean ± SD of 3 mice. NT, not tested, due to low cell numbers. Kruskal-Wallis test corrected for multiple comparisons using Dunn's test was used for statistical analyses. ^*^p < 0.05, ^**^p < 0.01, ^***^p < 0.001.

Short term-treatment consisting of 3 weekly injections with either ligand were sufficient to reduce the frequency of iNKT cells by around 80% or more in all organs analyzed (Figures 4A,B). However, TCR levels were not significantly reduced on α-GalCer-CD1d tetramer^+^ cells after ligand treatment (Figure 4C). iNKT cells in spleen and MLN of C26:0 and C20:2 treated mice had a lower expression level of NK1.1, while PD-1 was strongly upregulated on iNKT cells in the same organs compared to vehicle treated mice (Figures 4E,F). Interestingly, these markers on liver iNKT cells were less affected by the treatments. There was no *in vitro* proliferative response to the ligands by spleen cells from C26:0 treated mice, consistent with the decreased iNKT cell frequencies and anergic phenotype (35) after short-term C26:0 treatment, while the response by splenocytes from C20:2 and vehicle treated mice was comparable (Figure 4D). This indicates that the iNKT cells that remained in the spleen after C20:2 treatment were highly proliferative, considering their low frequency compared to that in vehicle treated mice. iNKT cells were not detectable in polyp and LP tissue after C26:0 treatment, and were very low after C20:2 treatment (Figure 4A). CD69 did not change significantly on iNKT cells in any organ after the treatments (Figure 4G). In contrast, the expression of the proliferation marker Ki67 was increased on iNKT cell in all organs of C20:2 treated mice, while a clear trend of increase in Ki67 in iNKT cells was seen in spleen and MLN after treatment with C26:0 (Figure 4H).

### Reduced Polyp Burden Was Associated With More Activated T Cells in Polyps and Decreased Frequency of Macrophages in Polyp and Spleen

**Figure 5:**
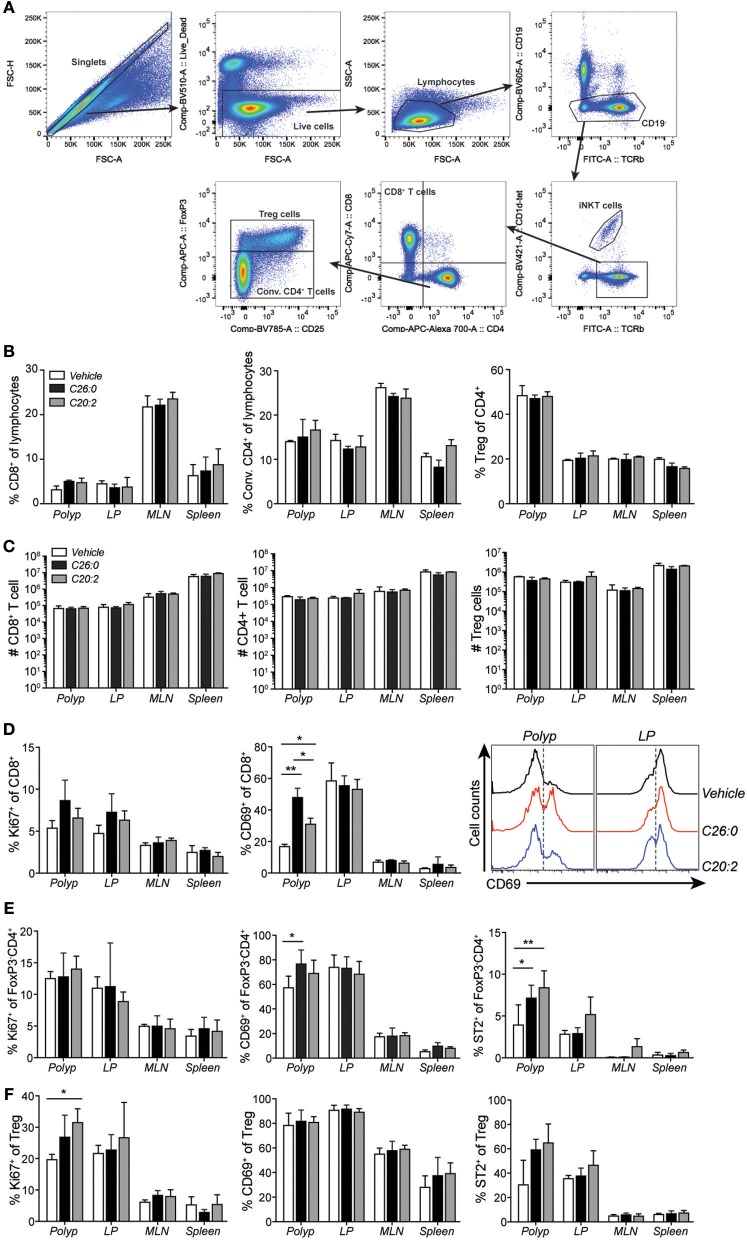
Short-term treatment with C26:0 and C20:2 increased the activation of polyp CD8^+^ T cells. **(A)** Gating strategies for CD8^+^ T cells (CD19^−^ α-GalCer-CD1d-tet^−^TCRβ^+^CD8^+^CD4^−^), conventional CD4^+^ T (Conv. CD4^+^ T, CD19^−^ α-GalCer-CD1d-tet^−^TCRβ^+^CD8^−^CD4^+^FoxP3^−^) cells, and regulatory T cells (Treg, CD19^−^ α-GalCer-CD1d-tet^−^TCRβ^+^CD8^−^CD4^+^FoxP3^+^) are shown for vehicle treated spleen. **(B)** Frequency of CD8^+^ T cells and Conv. CD4^+^ (CD4^+^FoxP3^−^) T cells among lymphocytes, and Treg cells (CD4^+^FoxP3^+^) among total CD4^+^ cells in treated *Apc*^*Min*/+^ mice in the indicated organs. **(C)** Absolute number of CD8^+^ T cells, Conv. CD4^+^ T cells, and Treg cells in the indicated organs of treated *Apc*^*Min*/+^ mice. **(D)** Expression of Ki67 and **(C)** CD69 on CD8^+^ T cells. **(E)** Expression of Ki67, CD69, and ST2 on Conv. CD4^+^ T cells. **(F)** Expression of Ki67, CD69, and ST2 on Treg cells. Data are presented as mean ± SD of 3 mice, and representative histograms are shown in **(D)**. Kruskal-Wallis test corrected for multiple comparisons using Dunn's test was used for statistical analyses. ^*^p < 0.05, ^**^p < 0.01.

**Figure 6:**
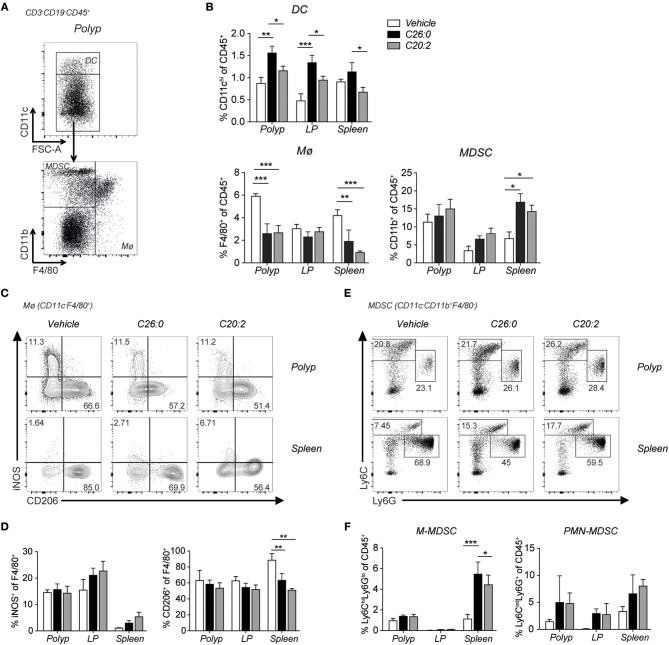
Short-term treatment with C26:0 and C20:2 reduced tumor infiltrating macrophages, and induced phenotypic M2 to M1 splenic macrophage switch in *Apc*^*Min*/+^ mice. Flow cytometry analysis was performed on innate immune cells in polyps, LP and spleen of treated mice. **(A)** Gating strategies for dendritic cells (DC, CD3^−^CD19^−^CD45^+^CD11c^hi^), macrophages (Mø, CD3^−^CD19^−^CD45^+^CD11c^lo/neg^ F4/80^+^), and myeloid-derived suppressor cells (MDSCs) (CD3^−^CD19^−^CD45^+^ CD11c^lo/neg^CD11b^+^) are shown for vehicle treated *Apc*^*Min*/+^ spleen. **(B)** Frequency of DCs, Mø and MDSCs among CD45^+^ cells in indicated tissues. **(C)** Representative staining of iNOS and CD206 expression on Mø. **(D)** Frequency of iNOS and CD206 expressing cells among macrophages in the same tissues. **(E)** Gating strategy for monocytic MDSC (M-MDSC) (CD3^−^CD19^−^ CD45^+^CD11c^lo/neg^ CD11b^+^Ly6c^hi^Ly6G^lo^) and polymorphonuclear MDSC (PMN-MDSC) (CD3^−^CD19^−^CD45^+^CD11c^lo/neg^CD11b^+^Ly6c^int^Ly6G^+^) shown for polyp and spleen. **(F)** Frequency of M-MDSC and PMN-MDSC among CD45^+^ cells in indicated tissues. Data are presented as mean ± SD of 3 mice. Kruskal-Wallis test corrected for multiple comparisons using Dunn's test was used for statistical analyses. ^*^p < 0.05, ^**^p < 0.01, ^***^p < 0.001.

We performed flow cytometry analysis of immune cells infiltrating polyps and compared them to cells in LP, MLN, and spleen (Figure 5). Frequencies and numbers of conventional CD4 T cells (CD4^+^ FoxP3^−^), CD8 T cells, or Treg cells (CD4^+^ FoxP3^+^) did not change by the treatments (Figures 5B,C). However, specifically in polyps, CD69 was up-regulated on CD8 T cells after both ligand treatments, and on CD4 T cells after C26:0 treatment (Figures 5A,B). Further, the IL-33 receptor ST2 was somewhat increased on polyp CD4 T cells after treatment. Ki67 was not significantly altered on T cells in any tissues analyzed except on Treg cells after C20:0 treatment (Figures 5D–F). Analysis of innate immune cells demonstrated a reduced frequency of tumor infiltrating macrophages after both ligand treatments (Figure 6). There was a reduction of macrophages also in the spleen where there was a shift of macrophage phenotype with slightly increased inducible nitric oxide synthase (iNOS) expression and significantly reduced CD206 levels, indicating a more pro-inflammatory phenotype after treatments (Figures 6B–D). Both treatments also increased the population of cells with a phenotype of myeloid derived suppressor cells (MDSC) (36), primarily of the monocytic type (Figures 6B,E,F). Taken together, this suggested that treatments associated with decreased polyp burden resulted in more activated CD4 and CD8 T cells and fewer macrophages in polyps, and further that there was a shift from anti-inflammatory toward pro-inflammatory macrophages in the spleen.

## Discussion

### Repeated Treatment With iNKT Cell Agonists Reduced iNKT Cell Numbers

Here we have investigated whether therapeutic activation of iNKT cells could suppress polyp development in the *Apc*^*Min*/+^ model for colorectal cancer. In this model, iNKT cells naturally promote polyp development associated with a suppression of TH1 gene expression, and an increase in M2 macrophages and Treg cells in the polyp tissue (28). We therefore expected that activation of iNKT cells by treatment with α-GalCer (C26:0), and more so the TH2 skewing derivative C20:2, might further enhance polyp growth. Consistently, we hypothesized that the TH1-biasing analog C-glycoside would strengthen TH1 anti-polyp immune responses and suppress polyp growth. To investigate this, *Apc*^*Min*/+^ mice were treated with C26:0, C20:2, C-glycoside or vehicle following two different treatment protocols. One protocol initiated treatment at 5 weeks of age (long-term treatment), around the time when polyps are initiated (30). The second protocol applied treatment from 12 weeks of age until sacrifice at 15 weeks of age (short-term treatment). This is during the time when the most extensive polyp growth occurs (28), a treatment period that would be closer to a clinical situation when initial polyps have been diagnosed. Weekly injections were selected as this protocol was previously shown to be effective in preventing development of type 1 diabetes (9). A brief summary of the results is shown in Table 1. Treatments with all three ligands using both protocols resulted in severely reduced iNKT cell populations systemically, and a phenotype consistent with activation (down regulation of NK1.1 and increased expression of Ki67) and anergy induction (upregulation of PD-1) (35). At the end of the treatment periods when mice were sacrificed, the α-GalCer-CD1d tetramer^+^ TCRβ^+^ populations were quite clearly separated from the rest of the cells in the FACS plots (Figures 2A, 4A). It therefore seems that remaining iNKT cells have not dramatically down-regulated TCR levels, however it cannot be excluded that some iNKT cells escaped detection due to reduced TCR expression. Compared to C26:0 and C-glycoside, splenic iNKT cells from mice treated with C20:2 were more proliferative in response to *in vitro* stimulation, consistent with a shorter duration of an anergic state of iNKT cells activated repeatedly with this ligand (37). These data are consistent with previous publications (16, 18). A brief summary of the results is shown in Table 1.

**Table 1:** Summary of effects by iNKT cell agonist treatments of *Apc*^*Min*/+^ mice.

	**Long term**			**Short term**		
	**Polyp #**	**Polyp size**	**iNKT loss/anergic phenotype**	**Polyp #**	**Polyp size**	**iNKT loss/anergic phenotype**
C26:0	↓^a^	↓	++^b^	≅^c^	↓	+++
C20:2	≅	↑	++	↓	↓	++
C-Glycoside	≅	≅	++	ND^d^	ND	ND

a *Arrows indicate increase or decrease of the polyp number and size*.

b *++/+++ was used to grade the iNKT cell loss/anergic phenotype*.

c *≅ Means no significant change*.

d *ND, not determined*.

### Long- and Short-Term Treatment With C26:0 Resulted in Reduced Polyp Burden

Treatment with C26:0 had a beneficial outcome, suppressing polyp numbers, as shown before (28) as well as polyp size in the long-term treatment. C26:0 also reduced polyp size using the short-term protocol, suggesting that C26:0 treatment enhanced the tumor immune response. Importantly, long-term treatment, but not short-term, significantly reduced polyp numbers also in colon. iNKT cells are present in LP of both SI and colon, however, the frequency of iNKT cells in colon is lower than in SI (38), which may explain the weaker tumor suppressive effect in colon by C26:0 treatment. Suppression of tumors by treatment with α-GalCer has been shown in several murine tumor models (39–42). The effect has been linked to production of IFN-γ by iNKT cells, activation, and IL-12 induction in DC and down stream activation of NK cells and other immune cells. Consistently, we demonstrate that C26:0 long-term treatment of *Apc*^*Min*/+^ mice induced polyp expression of genes associated with pro-inflammatory immunity, including *Ifng*. After short-term treatment, there was an increased activation of CD4 and CD8 T cells in polyps, together with a systemic reduction of macrophages and increased proportion of the pro-inflammatory M1 macrophage phenotype. It is well-known that activation of iNKT cells by α-GalCer results in down stream activation of other immune cells such as T cells, DC, and macrophages (43–45).

Activated macrophages can have divergent functions, and are often classified in a simplified scheme as having M1 or M2 phenotypes (46). M1 macrophages are associated with TH1 responses such as against intracellular pathogens and tumors, and produce IL-12 and large amounts of oxygen and nitrogen intermediates. M2 macrophages have high expression of mannose and scavenger receptors and produce IL-10 rather than IL-12, and promote TH2 immunity. Tumor infiltrating macrophages with an M2-like phenotype contribute to tumor progression by mechanisms that include suppression of T cell antitumor responses (47). It has been proposed that iNKT cells could contribute to reduced tumor burden indirectly by killing anti-inflammatory tumor associated macrophages (13). Taken together, this suggests that C26:0 treatment favored a TH1-type tumor immunity in *Apc*^*Min*/+^ polyps. This is consistent with described down-stream activation of diverse immune cells after α-GalCer administration and iNKT cell activation (9–12).

The underlying mechanism that changed the iNKT cell mediated immunosuppression in polyps of untreated *Apc*^*Min*/+^ mice to a TH1-like tumor microenvironment after C26:0 treatment was not investigated in further detail. It is possible that that C26:0 treatment of *Apc*^*Min*/+^ mice may alter the function of polyp iNKT cells rendering them more pro-inflammatory. Alternatively, the reduced frequencies of iNKT cells in *Apc*^*Min*/+^ polyps after treatment could have indirect pro-inflammatory effects as this may limit tumor immunosuppression by iNKT cells in polyps. While iNKT cells are generally regarded as protective in tumor immunity by enhancement of TH1 type immunity, previous publications have also demonstrated that iNKT cells can have natural tumor promoting effects in lymphomas (29). This was associated with suppression of anti-tumor CD8 T cells (48), reduced IFN-γ and elevated IL-13 production (49). iNKT cells can also be rendered unable to suppress melanoma metastasis, or will even enhance metastasis, after pretreatment with α-GalCer (33, 50), through the induction of IL-10 producing iNKT10 cells (51). Thus, iNKT cells can either have a beneficial, neutral or detrimental role in tumor immunity. A dual function is also well-established for the immune system at large in tumor immunity, which is influenced by inflammatory signals and complex interactions with cells in the tumor microenvironment shaping tumor immunity and immunosuppression (52).

### Long-Term Treatment Both With C-Glycoside or C20:2 Significantly Increased Polyp Burden Compared to C26:0

Surprisingly, long-term treatment with the TH1 skewing ligand C-glycoside significantly increased polyp numbers compared to C26:0 treatment, and even promoted the development of increased numbers of small SI polyps compared to vehicle treated mice. This is in contrast to publications demonstrating a more efficient anti-tumor effect of C-glycoside compared to C26:0 (16, 17). In these studies, C-glycoside induced prolonged stimulation of TH1 cytokine production and enhanced antimetastatic activity several-fold compared to α-GalCer. The divergent results may be due to a combination of distinct tumor models used and different administration protocols. In the studies by Tsuji and co-workers (16, 17), C-glycoside administration was performed once before challenge with B16 melanoma cells, while we have used a spontaneous orthotopic tumor model and repeated administration of lipids. Increased polyp burden after long-term treatment with C20:2 compared to both vehicle and C26:0 treatment was more anticipated, considering the preferential induction of TH2 associated cytokines previously demonstrated for this iNKT cell agonist. Analysis of gene expression in polyps of agonist and vehicle treated mice revealed that C-glycoside failed to induce the pro-inflammatory gene profile found in polyps from C26.0 treated mice. In contrast, *Il6, Ifng, Il1b, Il17a, Il17f*, and *Mmp3* transcripts were found at higher levels in polyps from C26:0 treated mice compared to polyps from mice treated with either C20:2 or C-glycoside, while increased expression of *Il4* and *Il10* was shared in the latter. This suggests that long-term treatment with C20:2 and C-glycoside may have induced an immunosuppressive microenvironment in the polyps. This is consistent with the capacity of C20:0 treatment to prevent autoimmune disease (19), but unexpected for the C-glucoside ligand.

### Long-Term and Short-Term Treatment With C20:2 Had Opposite Effects on Polyp Burden

It was expected that long-term treatment with the TH2 biasing ligand C20:2 would increase polyp growth, as evident by the increased numbers of large polyps compared to vehicle and C26:0 treated mice. However, the tumor suppressive effect after short-term treatment with C20:2 was unanticipated. Total polyp numbers were reduced, and there was a significant decrease in large polyps, compared to C26:0 and vehicle treated mice. Analysis of immune cells in polyps and other tissues after short-term C20:2 treatment demonstrated that, similar to C26:0 treatment, there was a significantly increased CD69 expression on CD8 T cells specifically in the polyps, accompanied by decreased proportion of macrophages in polyp and spleen. There was also a reduced ratio of macrophage expression of CD206 (an M2-associated mannose receptor) vs. iNOS, suggesting a shift from M2-like toward M1-like macrophage phenotype. Thus, this indicates that short-term treatment with both C26:0 and C20:2 enhanced a pro-inflammatory anti-tumor immune response. The tumor environment is known to strongly modulate the functions of tumor infiltrating immune cells. Such changes may also underlie the differential results obtained from long-term vs. short-term treatment with C20:2. It is possible that polyp infiltrating iNKT cells become affected by the polyp microenvironment and display altered functions at later stages. The unique phenotype and functional characteristics of polyp iNKT cells would be consistent with this notion (28). Alternatively, the downstream effects of iNKT cell activation with C20:2 at an early or late stage of disease may have diverse outcomes due to divergent functions in non-iNKT cells in the polyp microenvironment.

## Concluding Remarks

In the presented studies we were able to suppress intestinal polyp development in *Apc*^*Min*/+^ mice through the activation of iNKT cells using two different CD1d-restricted TCR ligands, α-GalCer (C26:0) and the synthetic analog C20:2, despite the natural polyp promoting role that iNKT cells display in the *Apc*^*Min*/+^ model. Taken together, the gene expression and immune cell analysis indicates that tumor reduction induced by iNKT cell ligand treatment was associated with more activated T cells in polyps, a switch toward M1-like macrophages and an enhanced pro-inflammatory immune environment. These data are consistent with results from *Apc*^*Min*/+^ mice lacking iNKT cells, where reduced polyp burden was similarly associated with increased T cell activation, a shift in macrophages from M2-like to M1-like phenotype, and increased expression of pro-inflammatory/TH1 associated genes (28).

Preclinical and clinical studies are continuing to improve the efficacy of iNKT cell targeted tumor immunotherapy though the development of altered ligands that induce desired immune response profiles, design of delivery strategies to prevent induced loss of iNKT cells or anergy, and to avoid side effects of the treatment, for example by approaches that localize the iNKT cell activation to the tumor site (13–15). Our results provide further support for the potential benefits of iNKT cell directed anti-tumor therapy in human cancer. However, the data also stress that agonist activation of iNKT cells using different ligands and different treatment schedules may lead to unpredictable results, and calls for caution when it comes to translating iNKT cell directed therapy to human disease.

## Author Contributions

YW, SS, and LL performed experiments. SS and YW assembled and analyzed data, and prepared figures. GB synthesized C26:0 and C20:2. SP contributed to experimental design and interpretation, and provided reagents. SC conceived the study and analyzed data. SC and YW wrote the paper, and all other authors critically reviewed the manuscript.

### Conflict of Interest Statement

The authors declare that the research was conducted in the absence of any commercial or financial relationships that could be construed as a potential conflict of interest.

## Abbreviations

α-GalCer: α-galactosylceramide Apc, adenomatous polypsis coli
IBD: inflammatory bowel disease
iNOS: inducible nitric oxide synthase
i.p.: intraperitoneal
LP: lamina propria
MDSC: myeloid derived suppressor cells
MLN: mesenteric lymph node
NKT: natural killer T
SI: small intestine
TCR: T cell receptor
Th: T helper.
