# Supplementary material for: Promotion or Suppression of Murine Intestinal Polyp Development by iNKT Cell Directed Immunotherapy
Source: Front Immunol. 2019 Mar 1;10:352. doi: 10.3389/fimmu.2019.00352 (PMC6405695; doi:10.3389/fimmu.2019.00352)
Supplement: Supplementary file 1 [file Data_Sheet_1.pdf]

Supplementary table

Table 1. CT values of RT2 profiler PCR array.

Gene Symbol	Vehicle	Vehicle	C26:0	C26:0	C20:2	C20:2	C-Glyc	C-Glyc
IL1B	28,756292	28,475857	25,225304	25,36706	28,19518	28,412233	28,487066	28,55132
IL1A	30,412743	30,529613	28,19476	28,83598	30,6095	30,33755	30,707672	31,005718
IL2	Undetermined	38,26219	Undetermined	Undetermined	Undetermined	Undetermined	Undetermined	Undetermined
IL4	32,999237	31,372097	33,347977	39,062935	33,136356	33,837204	32,976692	34,413044
IL5	39,23043	33,6469	33,516895	Undetermined	Undetermined	33,491558	Undetermined	38,099182
IL6	30,479329	30,776514	28,591154	28,957241	31,493366	32,794937	32,928616	32,382244
CXCL15	33,02147	33,353363	32,92533	34,778416	33,75106	33,482414	32,813892	32,9285
IL9	Undetermined	33,671936	Undetermined	Undetermined	Undetermined	34,15719	Undetermined	35,913883
IL10	34.83398753	34.895421	34,69388	34,68044	33,69825	33,26570858	34,81226	34,81226
IL11	29,42097	29,353106	26,705473	26,945166	28,54401	28,996088	29,828335	30,228884
IL12A	Undetermined	Undetermined	33,337082	33,399586	34,4298	34,173397	Undetermined	Undetermined
IL12B	Undetermined	39,410927	33,753536	32,81586	32,717106	32,6743231	Undetermined	Undetermined
IL13	31,842085	33,032738	31,489723	31,745598	30,87868	31,752813	31,720663	31,757524
IL15	28,383917	27,907545	29,211857	29,214058	29,452286	29,220793	29,77967	29,462957
IL17A	36,33212	34,15348	31,647736	31,241863	36,220264	35,74615	38,083477	38,60677
IL17F	33,637417	33,565838	30,893438	30,899405	35,495926	35,78361	35,198124	35,92216
IL18	25,766268	25,630117	24,970282	25,098763	26,689548	26,95382	26,439505	26,63358
IL21	Undetermined	34,420933	Undetermined	35,60441	Undetermined	Undetermined	Undetermined	Undetermined
IL22	34,485256	34,8328067	32,426437	31,360474	33,538082	33,142944	34,45153	Undetermined
IL22RA1	26,240137	26,224873	26,568974	26,67049	26,577402	26,730711	27,191095	27,262316
IL22RA2	Undetermined	38,03117	37,77455	38,220627	37,738125	Undetermined	39,847763	Undetermined
IL23A	33,96459	33,80621	29,777796	30,617178	30,87372	30,824888	31,363728	32,11385
IL25	34,407383	34,355736	35,35281	34,54698	Undetermined	Undetermined	Undetermined	Undetermined
CDC27	27,684994	27,39403	27,166338	27,070747	27,559616	27,725512	28,487455	28,291945
IL33	25,937637	25,835007	26,588541	26,900034	27,098099	26,839735	26,86932	27,085411

Gene Symbol	Vehicle	Vehicle	C26:0	C26:0	C20:2	C20:2	C-Glyc	C-Glyc
IFNg	30,98760033	30,81122398	28,88823318	28,63820472	30,754644	30,576418	29,83977127	29,22294235
TNF	30,795359	30,285654	29,249224	29,15396	30,131271	29,542618	30,517998	31,37853
TGFb1	26,890032	26,686434	25,70856	25,71503	26,802498	26,804222	27,578592	27,276085
IFNaR1	27,598568	27,318754	26,98256	26,872622	27,612373	27,693808	28,220697	28,209328
TSLP	32,50929	32,42403	31,724054	30,80993	31,731438	32,430344	31,719288	32,30949
IL4RA	26,532772	26,06632	25,623497	25,752794	26,34227	26,555344	26,945862	27,184937
IL13RA1	25,182747	25,088066	24,984539	24,887844	25,257635	25,356894	25,922962	25,955511
IL13RA2	33,50326	34,43644	31,244957	32,430923	32,97754	32,901337	35,39758	34,671883
CD4	31,57973	30,4197	30,241388	31,304182	31,002825	31,213253	31,803234	30,605864
CD8b1	33,63484	32,827442	32,674404	32,258495	33,19322	32,33977	34,370922	35,38704
CD19	31,59039	31,804253	28,163174	28,417292	30,398897	31,390055	32,30079	32,73269
FoxP3	32,16143	32,911777	33,32789	32,184578	33,88041	32,62542	32,55195	32,83147
Zbtb16	31,5577	30,24167	30,289492	30,322254	30,262058	30,00593	30,645723	30,680433
Arg1	30,533926	29,633171	26,503542	26,8762	26,672487	26,903288	28,482151	28,550856
Nos2	29,651196	29,109642	27,182579	27,336645	26,79684	27,00564	30,738335	30,667282
Chi3l3	29,894201	29,310398	31,15103	31,253824	33,79473	32,973335	32,98731	32,367554
Mrc1	29,250126	29,600237	28,210209	28,542425	28,940466	29,210398	30,3756	29,694578
Ly6G	26,455997	26,405209	24,90954	25,225233	26,887945	26,833326	27,683409	27,674894
Mpo	Undetermined	Undetermined	33,942858	33,238586	33,99356	33,353836	32,593575	32,69066
Rorc	27,956764	28,151085	27,598307	27,46759	28,299473	27,891037	28,707848	28,861465
Retnla	32,890366	32,702206	31,298792	32,761356	32,90297	33,541416	33,735447	34,385345
Tgm2	24,940754	24,864794	23,793768	23,91959	24,559937	24,53913	25,430607	25,607042
Ptgs2	28,96886	28,532776	26,827522	27,048637	28,47885	28,331656	30,373953	30,23925
Ido1	28,255823	28,10335	27,7363	27,759022	27,759148	27,888405	28,990368	28,997072
Tnfsf15	31,254896	30,537828	30,682365	30,519176	32,472607	31,066109	32,34465	32,178726
Nfkb1	26,166327	26,05817	25,853123	25,953289	26,591429	26,732256	26,80224	27,135323
Stat1	26,39652	25,90772	25,20166	25,266329	25,61116	25,865385	26,811684	26,961115
Stat3	25,524208	25,489908	24,949747	25,126305	25,71101	25,895391	26,466103	26,467018

Gene Symbol	Vehicle	Vehicle	C26:0	C26:0	C20:2	C20:2	C-Glyc	C-Glyc
Stat6	30,694674	29,552887	29,587952	29,513716	31,002575	30,950462	31,167347	31,516237
Tbx21	35,26522	33,74083	34,589104	34,76901	34,39661	33,35793	34,053303	33,556904
Gata3	31,302427	29,867495	30,341484	30,768564	30,987955	32,046173	30,746717	31,90108
Egf	33,60048	31,78592	31,314182	30,79533	32,787809	32,891468	32,222046	34,736362
Egfr	28,027485	27,836527	27,714989	27,98199	28,354923	28,508741	28,738182	28,542099
Myc	26,51441	26,201715	25,855612	26,329304	26,529163	26,932564	26,844877	26,949593
MAPK1	25,158848	25,134863	24,846367	24,985151	25,47681	25,508629	25,98779	26,112783
Fgf2	31,843287	30,997429	30,295738	31,755142	31,25592	31,417881	31,458136	31,884989
CXCR2	32,590328	32,371338	30,16069	29,990852	33,512154	31,6788	32,547234	33,494747
CXCR3	32,02927	31,21974	30,588305	30,470844	31,47457	32,92831	33,17204	33,038933
CCR2	31,564692	31,50823	30,168337	30,184275	32,64135	31,99914	31,310474	31,282812
CCR6	32,517357	30,793005	28,267395	28,700409	30,584871	30,665234	31,130674	30,95688
CCL20	31,758171	32,35291	29,852388	30,43387	34,711723	32,423798	31,383295	31,378145
CXCL1	30,627308	31,023197	27,02234	27,120844	29,618364	29,872156	30,844826	31,175365
CXCL10	29,11982	28,681377	26,831848	27,118189	28,18063	27,953741	29,238398	29,935173
CXCL9	33,801567	33,651733	30,165598	29,736889	32,240814	31,49455	33,92944	32,977425
CXCL11	31,802334	32,126205	36,407566	33,967873	35,35689	37,17091	36,45453	37,45692
Prph	31,875235	31,633768	30,632002	31,365768	31,601454	32,17125	31,790028	32,52245
Gzma	24,643274	24,429914	25,104734	25,150684	24,79831	24,6526	24,595722	24,882496
Gzmb	26,9381	27,227303	27,624332	28,341473	27,535835	27,354956	28,024317	28,405613
Klrk	33,172737	34,51918	31,951693	32,235462	32,83034	31,837646	34,25888	34,90986
Rae1	26,485619	26,396757	25,845715	27,306517	26,915442	26,943377	27,132294	27,559755
H60a	Undetermined	Undetermined	Undetermined	Undetermined	Undetermined	Undetermined	39,007385	39,088234
Bcl2l1	26,374437	26,415997	25,735851	25,936024	26,411018	26,321903	27,259096	27,295906
XIAP	26,875235	27,126915	26,647385	26,709187	26,889328	27,092838	27,806337	27,834162
VEGFA	25,750013	25,85764	25,77467	25,815027	26,314173	26,178308	26,64494	26,756073
MMP9	27,512754	27,64799	26,221966	26,102495	27,962254	27,699192	28,27853	28,304295
MMP3	29,269575	29,704872	26,66867	26,919802	30,636618	31,313356	30,281519	30,368116

Gene Symbol	Vehicle	Vehicle	C26:0	C26:0	C20:2	C20:2	C-Glyc	C-Glyc
MMP1a	31,965881	32,15629	30,497587	31,477795	31,443045	31,808681	32,649002	33,181423
CD274	29,400713	30,156973	28,802444	28,812069	29,315514	28,741144	30,714323	31,555237
PDCD1lg2	33,907276	30,833748	32,005486	31,147188	30,880981	31,274351	31,345255	31,33355
PDCD1	34,85285	33,29968	31,253504	31,463833	31,377253	33,58344	32,660355	32,000416
PDGFB	30,525467	30,395912	29,152092	29,505192	30,159779	30,231392	30,91851	31,248661
MGDC[1]	Undetermined	Undetermined	Undetermined	Undetermined	Undetermined	Undetermined	Undetermined	Undetermined
PPC[2]	19,200138	19,51159	18,919792	19,197117	19,170702	18,940636	18,942568	19,209784
OOC[3]	19,010017	19,403076	18,923132	19,175781	19,223211	19,354311	19,18461	19,164968
GUSB[3]	26,767357	26,687962	25,447054	25,531473	26,755095	26,59857	26,99921	27,13687
HSP90ab1[3]	21,158514	21,182281	20,532156	20,672918	21,670448	21,692484	21,974	22,178633
Actb[3]	20,175625	19,857176	19,593216	19,813177	20,180254	20,296507	20,671597	20,813639
GapDH[3]	21,375334	21,25716	21,149784	21,292528	21,932728	22,064014	22,339834	22,44082
Rn18s[3]	16,612576	16,744986	16,382978	16,340029	15,964559	15,957885	17,203585	17,311443
Hprt[3]	31,318022	30,55488	29,573097	30,818283	30,754593	31,518929	30,81823	31,75425
Ppl32[3]	21,085623	21,178741	20,170324	20,241163	20,374191	20,391272	20,577023	20,665985

4

[1] MGDC stands for Mouse Genomic DNA Control, which was used as negative control.
[2] PPC stands for PCR Positive Control.
[3] Reference genes used for the PCR array.
